# Role of FDG-PET/CT in prediction of pathological tumor response and survival after trimodality therapy for esophageal squamous cell carcinoma

**DOI:** 10.1186/1749-8090-10-S1-A244

**Published:** 2015-12-16

**Authors:** Yoichi Hamai, Jun Hihara, Takaoki Furukawa, Ichiko Yamakita, Tomoaki Kurokawa, Morihito Okada

**Affiliations:** 1 Department of Surgical Oncology, Research Institute for Radiation Biology and Medicine, Hiroshima University, Hiroshima, 734-8551, Japan

## Background/Introduction

Trimodality therapy has frequently been applied to patients with locally advanced esophageal cancer. However, to preoperatively predict the response to neoadjuvant therapy and prognosis is difficult.

## Aims/Objectives

The present study aimed to determine the diagnostic applicability of 18F-fluorodeoxyglucose-positron emission tomography/computed tomography (FDG-PET/CT) to evaluating the response and prognosis after trimodality therapy for esophageal squamous cell carcinoma (ESCC).

## Method

The responses of 111 patients with ESCC who underwent neoadjuvant chemoradiotherapy (nCRT) followed by surgery were monitored by measuring the maximal standardized uptake value (SUVmax) of the primary tumor on FDG-PET before and after nCRT. Associations between SUVmax and pathological response of primary tumor and prognosis were analyzed.

## Results

We compared the SUVmax between good (Japan Esophageal Society response evaluation criteria grades 3/2; n = 87, 78.4 %) and poor (grade 1; n = 24, 21.6 %) responders. The SUVmax after nCRT (post-SUVmax) in good and poor responders were 2.7 ± 0.9 and 4.4 ± 2.2, respectively (p < 0.0001). The rates of the SUVmax decrease after nCRT (ΔSUVmax) in these patients significantly differed (71 ± 14% vs. 60 ± 21%, respectively, p = 0.003). The area under receiver operating characteristic curves (AUC) showed that the optimal cut-off for post-SUVmax and ΔSUVmax were 3.7 (AUC, 0.76; 95% CI, 0.63-0.89; P < 0.001) and 70% (AUC, 0.65; 95% CI, 0.52-0.78; P = 0.02) for predicting good responder, respectively, and that the patients could be separated into groups with and without good response using these cut-off values. The 5-year overall survival rate was significantly higher for patients with ΔSUVmax > 70% than ≥ 70 % (66% vs. 42%, p = 0.04), and multivariable analysis including preoperative factors also revealed ΔSUVmax (≥ 70/> 70%) as an independent prognostic factor for disease-specific survival (OR, 2.22; 95%CI, 1.02 - 4.76; p = 0.04).

## Discussion/Conclusion

Post-SUVmax and ΔSUVmax were valuable preoperative factors for predicting tumor response and survival in patients with ESCC after trimodality therapy. Thus, FDG-PET/CT findings are useful for tailoring optimal therapies for individual patients with ESCC.

